# Supplementary material for: Phenotypic Screening Identifies Modulators of Amyloid Precursor Protein Processing in Human Stem Cell Models of Alzheimer’s Disease
Source: Stem Cell Reports. 2017 Mar 9;8(4):870–82. doi: 10.1016/j.stemcr.2017.02.006 (PMC5390098; doi:10.1016/j.stemcr.2017.02.006)
Supplement: Document S1. Supplemental Experimental Procedures and Figures S1 and S2 [file mmc1.pdf]

Stem Cell Reports, Volume *8*

Supplemental Information

Phenotypic Screening Identifies Modulators of Amyloid Precursor Protein Processing in Human Stem Cell Models of Alzheimer's Disease

Philip W. Brownjohn, James Smith, Erik Portelius, Lutgarde Serneels, Hlin Kvartsberg, Bart De Strooper, Kaj Blennow, Henrik Zetterberg, and Frederick J. Livesey

Supplemental Information

Supplemental Data

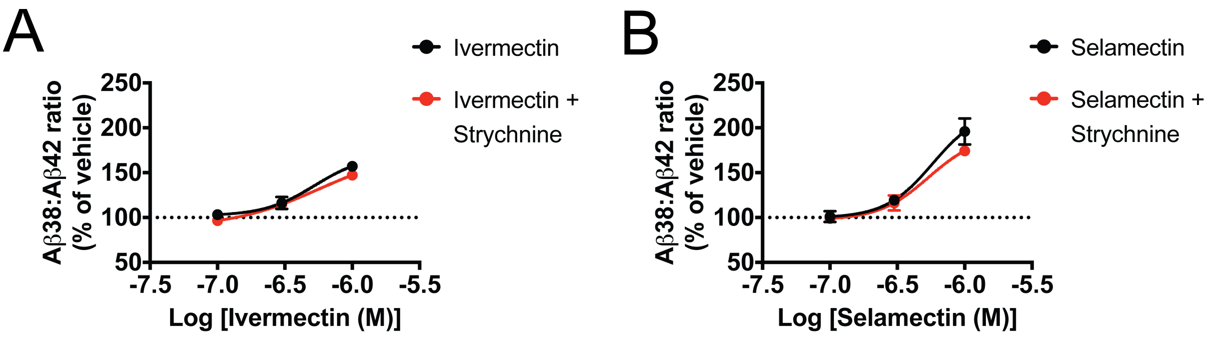

Figure S1. Avermectins do not alter Aβ production in human neurons via activation of glycine receptors. Ivermectin (A) and selamectin (B) dose dependently increase the ratio of Aβ38 to Aβ42 in the presence and absence of the glycine receptor antagonist strychnine (30 μM). n = 3 cultures/treatment. Error bars represent standard deviations.

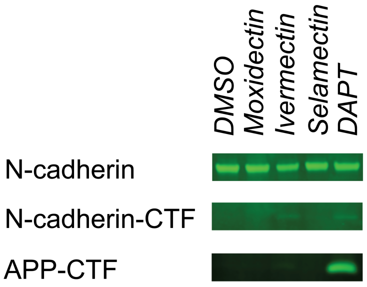

Figure S2. Chronic treatment with avermectins does not cause significant accumulation of γ-secretase substrate CTFs in human neurons. Treatment of human neurons for 30 days with DAPT results in mild accumulation of N-cadherin-CTF, and significant accumulation of APP-CTF. In contrast, 30 days of treatment with moxidectin or the avermectins ivermectin and selamectin causes no significant accumulation of N-cadherin-CTF or APP-CTF, while ivermectin results in mild accumulation of N-cadherin-CTF. Western blots are representative of 2 – 3 cultures/treatment.

Supplemental Experimental Procedures

Cell-free γ-secretase assays

In vitro reactions with 150 ng/μl DRM γ-secretase from Sf9 cells expressing human PSEN1-APH1A-NCT-PEN2 γ-secretase and 1.2 μM APP-C99–3×FLAG were performed in 0.3% CHAPSO and 2.5% DMSO or compound at the indicated concentration for 1 h at 37 °C. To detect AICD the substrate was extracted by adding 1 volume chloroform/methanol (2:1, v/v). Then, the aqueous fraction (AICD Products) was taken and subjected to SDS-PAGE and quantitative western immunoblot. AICD-3×FLAG was determined with the anti-FLAG M2 (Sigma) and goat anti-mouse IR800 (Pierce) antibodies. Infrared signals were detected and quantified using the Odyssey Infrared Imaging System. To determine de novo production of Aβ peptides, Aβ38, Aβ40, and Aβ42 levels in reactions were quantified on Multi-Spot 96-well plates precoated with anti-Aβ38, -Aβ40, and -Aβ42 antibodies and detected with SULFO-TAG JRF/AβN/25 using multiplex MSD technology (Meso Scale Discovery). The anti-Aβ antibodies were generously provided by Mark Mercken from Janssen Pharmaceutica.

Statistical analysis

Normality was probed with the Shapiro-Wilk normality test and by visualization of Q-Q plots. Dose-response data were analyzed with parametric one-way ANOVA when normally distributed, or Kruskall-Wallis H test when not. For antagonism studies, data were analyzed by two-way ANOVA. While small deviations from

normality could not be rectified with transformation in this case, parametric analysis was still performed in order to obtain the most statistical power, with the caveat that the type-I error rate was likely to be inflated. Cell-free assays were analyzed by corrected one-sample t-tests or Wilcoxin signed rank tests. Multiple testing was corrected using the Holm-Šídák method where appropriate.
