# Supplementary material for: Phenotypic Screening Identifies Modulators of Amyloid Precursor Protein Processing in Human Stem Cell Models of Alzheimer’s Disease
Source: Stem Cell Reports. 2017 Mar 9;8(4):870–82. doi: 10.1016/j.stemcr.2017.02.006 (PMC5390098; doi:10.1016/j.stemcr.2017.02.006)
Supplement: Document S2. Article plus Supplemental Information [file mmc2.pdf]

Stem Cell Reports
Article
ISSCR
OPEN ACCESS

Phenotypic Screening Identifies Modulators of Amyloid Precursor Protein Processing in Human Stem Cell Models of Alzheimer's Disease

Philip W. Brownjohn,[1] James Smith,[1] Erik Portelius,[2,3] Lutgarde Serneels,[4,5] Hlin Kvartsberg,[2,3] Bart De Strooper,[4,5,6] Kaj Blennow,[2,3] Henrik Zetterberg,[2,3,6] and Frederick J. Livesey[1,*]
[1]The Gurdon Institute and Department of Biochemistry, University of Cambridge, Cambridge CB2 1QN, UK
[2]Department of Psychiatry and Neurochemistry, Institute of Neuroscience and Physiology, The Sahlgrenska Academy at the University of Gothenburg, 431 80 Mölndal, Sweden
[3]Clinical Neurochemistry Laboratory, Sahlgrenska University Hospital, 431 80 Mölndal, Sweden
[4]VIB Center for the Biology of Disease, 3000 Leuven, Belgium
[5]Center for Human Genetics (CME), Leuven Research Institute for Neuroscience and Disease (LIND), University of Leuven (KU Leuven), 3000 Leuven, Belgium
[6]Department of Molecular Neuroscience, UCL Institute of Neurology, Queen Square, London WC1N 3BG, UK
*Correspondence: rick@gurdon.cam.ac.uk
http://dx.doi.org/10.1016/j.stemcr.2017.02.006

SUMMARY

Human stem cell models have the potential to provide platforms for phenotypic screens to identify candidate treatments and cellular pathways involved in the pathogenesis of neurodegenerative disorders. Amyloid precursor protein (APP) processing and the accumulation of APP-derived amyloid β (Aβ) peptides are key processes in Alzheimer's disease (AD). We designed a phenotypic small-molecule screen to identify modulators of APP processing in trisomy 21/Down syndrome neurons, a complex genetic model of AD. We identified the avermectins, commonly used as anthelmintics, as compounds that increase the relative production of short Aβ peptides at the expense of longer, potentially more toxic peptides. Further studies demonstrated that this effect is not due to an interaction with the core γ-secretase responsible for Aβ production. This study demonstrates the feasibility of phenotypic drug screening in human stem cell models of Alzheimer-type dementia, and points to possibilities for indirectly modulating APP processing, independently of γ-secretase modulation.

INTRODUCTION

As the burden of neurodegenerative disease on an aging population increases, it is striking to note that there remain no approved disease-modifying treatments for dementia. Drug discovery in this area has been challenging, as while there has been undeniable progress, our understanding of the biology and mechanisms underpinning these complex conditions still remains limited. The advent of reprogramming technology in human cells (Takahashi et al., 2007; Takahashi and Yamanaka, 2006) has enabled the generation of patient-derived neurons from accessible somatic cells of individuals carrying genetic forms of neurodegenerative diseases. The ability to recapture pathological processes in disease-affected neuronal types derived from individuals with genetic forms of disease has been demonstrated in a number of conditions, including motor neurons in spinal muscular atrophy (Ebert et al., 2009), dopaminergic midbrain neurons in Parkinson's disease (Sanchez-Danes et al., 2012), and cortical neurons in Alzheimer's disease (AD) (Israel et al., 2012; Shi et al., 2012b; Yagi et al., 2011). Using cellular phenotypes reproduced in appropriate human cell types, it is now possible not only to study fundamental disease biology but also to identify disease-modifying pathways using genetic or pharmacological phenotypic screening in a relevant biological context. We report here a phenotypic screen of small molecules in cortical neurons with a genetic form of AD, performed with the aim of identifying compounds modifying the production of amyloid β (Aβ), an aggregation-prone and toxic peptide central to AD pathology.

The amyloid cascade hypothesis of AD proposes that accumulation and deposition of Aβ peptides, derived from the proteolytic processing of amyloid precursor protein (APP), is central to the development of AD (Hardy and Allsop, 1991; Hardy and Higgins, 1992). While AD commonly presents sporadically later in life (sAD), evidence for the amyloid hypothesis was drawn from observations that rare autosomal dominant missense mutations in the genes encoding APP, or presenilin 1 (PSEN1) or 2 (PSEN2), which form the catalytic region of the γ-secretase complex responsible for proteolytic cleavage of APP into Aβ peptides, lead to highly penetrant familial forms of early-onset AD (fAD) (Ertekin-Taner, 2007). Increased expression of the *APP* gene, due either to duplication of the *APP* locus (APP_{dup}) (Rovelet-Lecrux et al., 2006; Sleegers et al., 2006), or trisomy of chromosome 21 (Rumble et al., 1989)—containing the *APP* gene—in Down syndrome (trisomy 21 [TS21]), also leads to increased production and accumulation of Aβ peptides and the early onset of AD pathology. While the

 © 2017 The Authors.
This is an open access article under the CC BY license (http://creativecommons.org/licenses/by/4.0/).

mechanistic link between Aβ accumulation and neuronal dysfunction in both fAD and sAD remains unclear, targeting the processing of APP and the production of Aβ within the framework of the amyloid hypothesis has remained an attractive approach for AD drug discovery in recent years.

It has been proposed that the dynamic balance between longer, more toxic Aβ peptides, in particular the 42-amino-acid Aβ42, and shorter Aβ peptides is a more significant determinant of disease initiation and progression than total Aβ production (Findeis, 2007; Kuperstein et al., 2010). This suggests that modulating rather than inhibiting processing may be an effective strategy while avoiding adverse effects due to altered proteolysis of other substrates of γ-secretase. After initial β-secretase cleavage, the remaining membrane-bound fragment of APP is subjected to endopeptidase and then stepwise carboxypeptidase cleavage by γ-secretase, generating progressively shorter Aβ peptides (Takami et al., 2009). By augmenting the carboxypeptidase efficiency of γ-secretase with γ-secretase modulators (GSMs), it is possible to shift the production of Aβ peptides away from longer more toxic species toward shorter forms, without affecting total Aβ production or γ-secretase targeting of other substrates. While the results of larger clinical trials are yet to be reported, GSMs have demonstrated target engagement in patients (Soares et al., 2016; Toyn et al., 2016; Yu et al., 2014) and remain a promising avenue for development.

APP has a complex life cycle: in addition to its processing by β- and γ-secretase, it undergoes proteolytic turnover in a number of different cellular compartments by numerous different proteases (Small and Gandy, 2006). This complexity suggests that it may be possible to alter amyloidogenic APP processing in a secretase-independent manner to shift Aβ peptide production toward shorter forms, at the expense of production of longer, toxic peptides. The aim of this study was to ask whether it was possible to identify secretase-independent, small molecule modulators of Aβ processing that would shift the production of Aβ fragments in human cortical neurons away from Aβ42 to shorter, non-toxic forms. To do so, we performed a small-molecule phenotypic screen in TS21 cortical neurons, which we have previously shown to produce highly elevated levels of Aβ peptides (Shi et al., 2012b). Using this approach, we identified a family of macrocyclic lactone anthelminthic compounds, the avermectins, which reproduce the effects of GSMs, without acting directly on the γ-secretase complex or causing accumulation of γ-secretase substrates. These data demonstrate that phenotypic screening in human stem cell models of AD provides a potentially powerful strategy for identifying disease-modifying pathways and compounds, independent of known approaches to modulating APP processing.

RESULTS

A Primary Phenotypic Screen Identifies Modifiers of Aβ Production

To identify small-molecule modifiers of Aβ production in human neurons, we performed a phenotypic screen in cortical neurons differentiated from TS21 induced pluripotent stem cells (iPSCs) cultured in 96-well plates (Figure 1). Neurons derived from this genetic background overproduce all Aβ peptides (Shi et al., 2012b), and thus provide a sensitized background for drug screening in this context. A single-point screen of the Prestwick Chemical library was performed at 1 μM, with drugs and media refreshed at 48-hr intervals. Extracellular medium collected after 4 days of drug treatment was analyzed by multiplexed immunoassay to assess concentrations of Aβ38, Aβ40, and Aβ42. The activity of lactate dehydrogenase (LDH) in extracellular medium collected after 6 days of treatment was used as an indicator of cellular toxicity. The aim of this study was to identify compounds that shift APP processing away from the production of longer, potentially toxic forms of Aβ, specifically Aβ42, as indicated by increases in the ratio of Aβ38/Aβ42 and/or Aβ40/Aβ42.

To evaluate the assay performance within each plate, we calculated the coefficient of variation (CV) of DMSO controls (n = 5 cultures/plate) for each outcome measure. Mean CVs for Aβ38/Aβ42 and Aβ40/Aβ42 ratios in DMSO control-treated cultures were 9.12% and 7.58%, respectively (Figures 1C and 1E). These results indicate a low level of variation for calculated Aβ ratios in control conditions, and a stable platform for the identification of hit compounds.

A control-independent method, implemented using the open-source Bioconductor cellHTS2 package (Boutros et al., 2006), was used to identify hit compounds, with the B-score adjustment used to correct for positional differences within the 96-well plates (Brideau et al., 2003). After excluding 73 compounds for increased LDH activity (B score >3), 55 compounds were identified that reduced the relative contribution of Aβ42 to total Aβ production (B score >3 for increased Aβ38/Aβ42 ratio and/or Aβ40/Aβ42 ratio). A validation screen was subsequently performed whereby hit compounds were tested again at 1 μM in triplicate, and their ability to reproduce their initial effect was confirmed either by a significant Fisher's least significant difference test or at least two of three replicates reproducing a >10% increase in the Aβ38/Aβ42 or Aβ40/Aβ42 ratio compared with DMSO treatment. Validated hits were tested for dose response, and two compounds

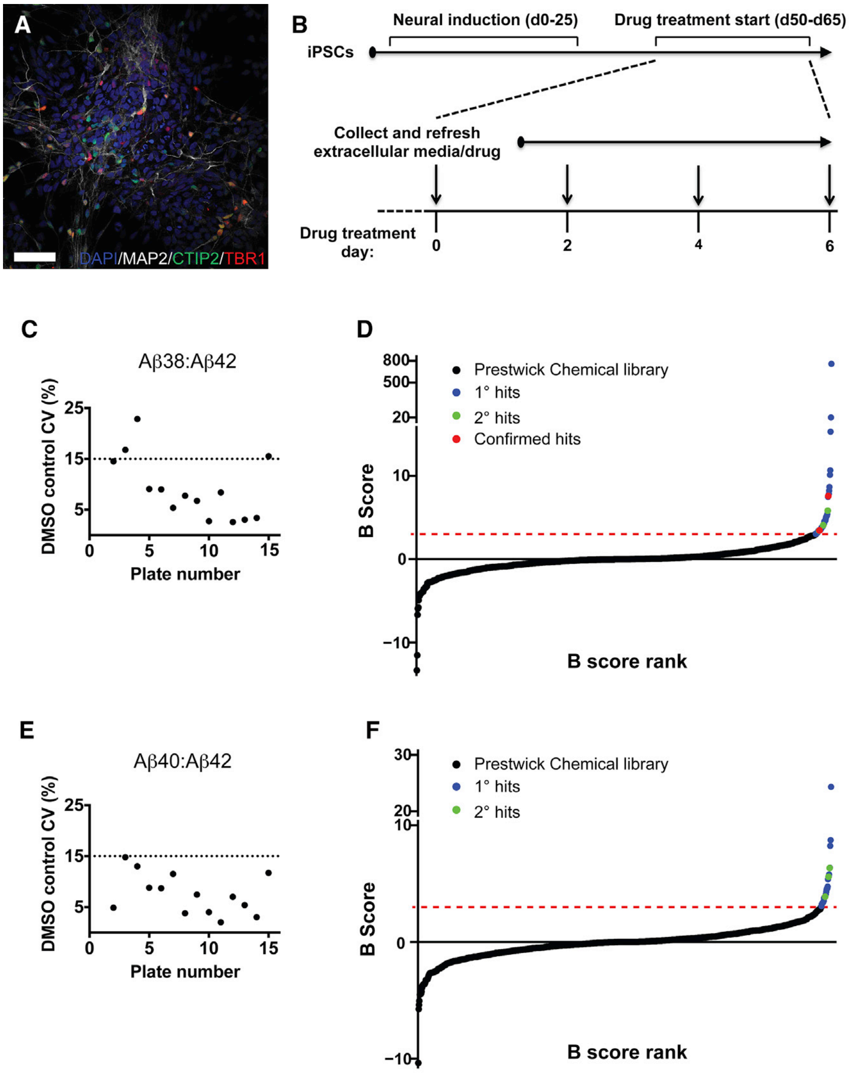

Figure 1. Phenotypic Screen of the Prestwick Chemical Library for Modifiers of Aβ Production in TS21 Cortical Neurons

(A) Fifty days after the initiation of neural induction, cortical neurons differentiated from TS21 iPSCs express microtubule-associated protein 2 (MAP2), and the cerebral cortex neuronal markers CTIP2 and TBR1. Scale bar represents 50 μm.

(B) Timeline of the primary screen of the Prestwick Chemical library in TS21 iPSC-derived cortical neurons. d, day.

(C and E) Coefficients of variation (CV) for vehicle (DMSO)-treated cultures for (C) Aβ38/Aβ42 and (E) Aβ40/Aβ42 ratios indicate a stable and sensitive platform for identifying hit compounds (dashed line represents recommended upper limit of 15% [Inglese et al., 2007]).

(D and F) A primary screen of the Prestwick Chemical library (black symbols) identified a number of primary hits (blue symbols) altering the Aβ38/Aβ42 (D) and Aβ40/Aβ42 (F) ratios (dashed line represents B-score >3). Primary hits were validated in a secondary screen (green symbols) and then confirmed with a dose response (red symbols).

were confirmed as hits based upon their ability to increase the ratio of Aβ38 to Aβ42 in a dose-dependent manner (Figure 1D). This resulted in an overall hit rate of 0.167% from the Prestwick Chemical library.

Avermectins Alter the Aβ38/Aβ42 Ratio in Human Cortical Neurons

In TS21 cortical neurons, we found that the previously described non-steroidal anti-inflammatory drug (NSAID)-derived GSM (R)-flurbiprofen (Figure 2A) and the imidazole-based GSM E2012 (Figure 2B) both increased the Aβ38/Aβ42 ratio in a dose-dependent manner ($F_{(7,\ 16)}$ = 43.77, $p < 0.0001$; $F_{(7,\ 16)} = 1633$, $p < 0.0001$, respectively). In a manner analogous to the known GSMs, one compound identified in the primary screen, abamectin (Figure 2C), demonstrated a marginal increase in the ratio of Aβ38 to Aβ42 ($F_{(5,\ 15)} = 2.754$, $p = 0.0586$), while a structurally related compound in the library, ivermectin (Figure 2D), significantly increased the Aβ38/Aβ42 ratio in a dose-dependent manner ($F_{(5,\ 15)} = 4.435$, $p = 0.0221$). These two compounds are members of the avermectin chemical class of macrocyclic lactones, and have been used for decades as anthelmintics to treat parasitic infections in animals and humans, but do not efficiently cross the blood-brain barrier (Fisher and Mrozik, 1992).

To determine whether the effect on the Aβ38/Aβ42 ratio was a common property across the avermectin family, we assessed two related compounds. Emamectin benzoate (Figure 2E) had a similar, moderate effect ($F_{(5,\ 15)} = 6.284$, $p = 0.0050$), whereas selamectin (Figure 2F) exhibited higher potency on the Aβ38/Aβ42 ratio ($F_{(5,\ 15)} = 16.18$, $p = 0.0003$). The effect appears to be specific to the avermectin family, as moxidectin (Figure 2G), a member of the closely related milbemycin class of macrocyclic

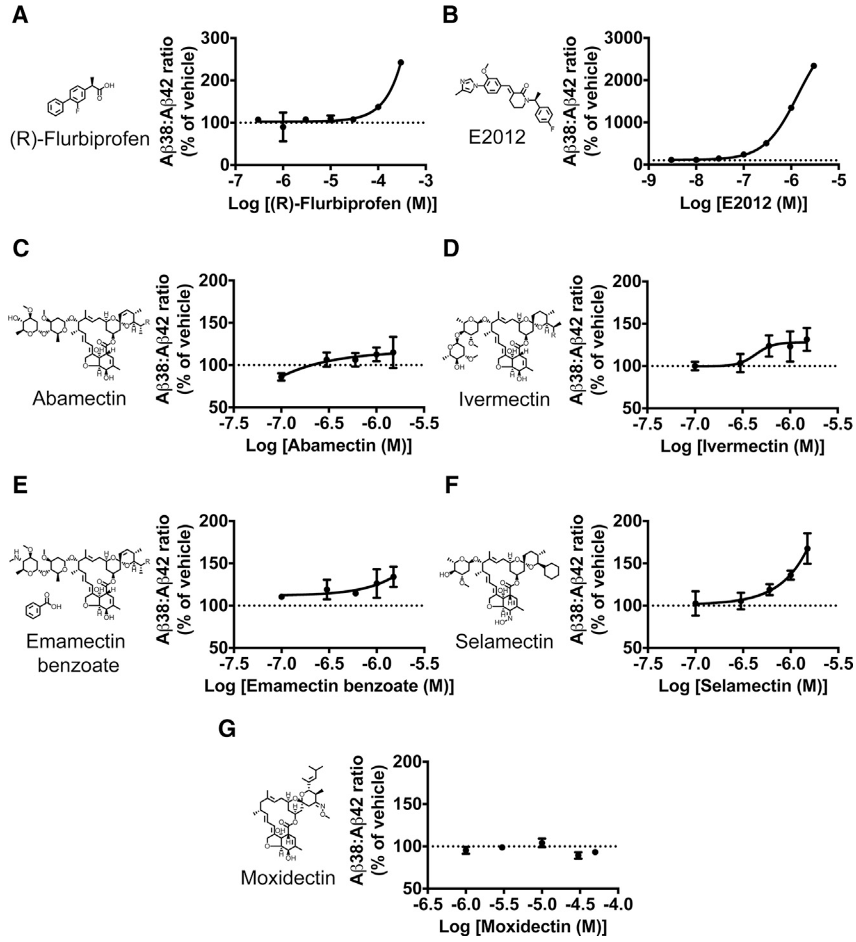

Figure 2. Avermectins Increase the Aβ38/Aβ42 Ratio in Human TS21 Neurons, in a Manner Phenotypically Similar to Previously Identified γ-Secretase Modulators

The γ-secretase modulators (R)-flurbiprofen (A) and E2012 (B) dose-dependently increase the ratio of Aβ38 to Aβ42 in human TS21 cortical neurons. The avermectins abamectin (C) and ivermectin (D), identified in the primary screen, as well as emamectin benzoate (E) and selamectin (F), also dose-dependently increase the Aβ38/Aβ42 ratio, while the structurally related milbemycin moxidectin (G) has no consistent effect. n = 3–6 cultures/concentration. Error bars represent SD. Note that abamectin, ivermectin, and emamectin benzoate are all mixtures of B1a ($R = CH_2CH_3$) and B1b ($R = CH_3$) components.

lactones, had no consistent effects on this ratio, despite its greater water solubility allowing for much higher concentrations to be achieved ($H_{(5)} = 11.77$, $p = 0.0380$; however, for all post hoc comparisons $p > 0.05$).

All avermectins increased the Aβ38/Aβ42 ratio by increasing Aβ38 and/or reducing Aβ42 (Figures 3A and 3C), while some had additional effects on Aβ40 (Figure 3B). Abamectin, ivermectin, and selamectin all caused a significant increase in Aβ38 ($F_{(5, 15)} = 6.543$, $p = 0.0040$; $F_{(5, 15)} = 13.05$, $p = 0.0003$; $H_{(5)} = 16.59$, $p = 0.0053$, respectively), while emamectin benzoate and selamectin caused a significant decrease in Aβ42 ($F_{(5, 15)} = 5.889$, $p = 0.0033$; $F_{(5, 15)} = 7.22$, $p = 0.0039$, respectively). In addition, abamectin, ivermectin, and selamectin all caused a significant decrease in Aβ40 ($F_{(5, 15)} = 6.063$, $p = 0.0058$; $H_{(5)} = 12.35$, $p = 0.0303$; $F_{(5, 15)} = 53.38$, $p = 0.0003$, respectively).

As macrocyclic lactones have a high lipophilicity, we considered whether the effects of avermectins on Aβ production were due to non-specific perturbations of the membrane and/or γ-secretase-APP interaction. Previously reported cLogP values (Prichard et al., 2012) indicate no correlation between lipophilicity and efficacy for the compounds studied: the largely inactive milbemycin moxidectin and the most potent avermectin selamectin share comparable cLogP values of 6 and 6.3, respectively, while the moderately active avermectins abamectin and ivermectin have reported cLogP values of 5.3 and 4.8, respectively (Prichard et al., 2012). Given that these observations strongly suggest that the modulation of Aβ production by the avermectins is not a simple function of their relative lipophilicity, we performed a number of experiments to identify possible molecular targets.

Avermectins Have Complex Effects on APP Processing and Aβ Peptide Production

The Aβ38/40/42 immunoassay captures only a fraction of Aβ peptides secreted from human neurons. As the analysis of these three peptides indicated that the avermectins exhibit complex effects on APP proteolysis, extracellular medium was analyzed with an approach combining immunoprecipitation and matrix-assisted laser desorption/ionization (IP-MALDI) to assess a wider spectrum of Aβ peptides.

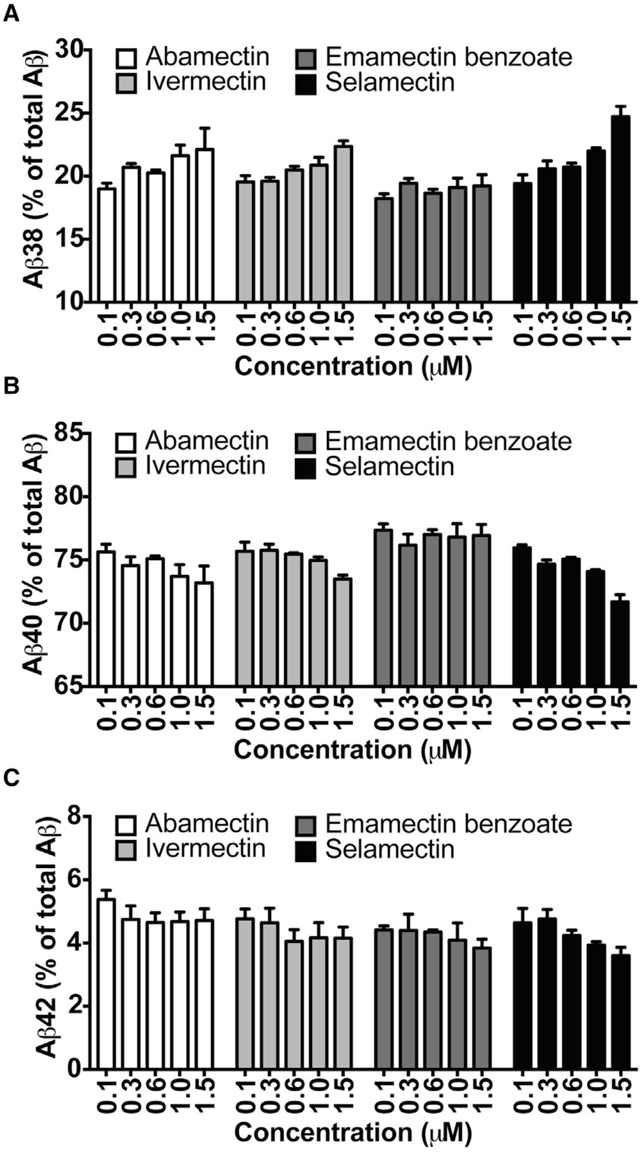

Figure 3. The Avermectin-Induced Increase in the Aβ38/Aβ42 Ratio Is Driven by an Increase in Aβ38 and/or a Decrease in Aβ42

(A and C) When the contribution of each Aβ species is considered as a percentage of total as detected by immunoassay, avermectins cause a dose-dependent increase in Aβ38 (A) and/or a decrease in Aβ42 (C).

(B) With the exception of emamectin benzoate, the avermectins also cause a dose-dependent decrease in Aβ40 levels (B).

n = 3–6 cultures/concentration. Error bars represent SD.

It has previously been shown that Aβ1-14, Aβ1-15, and Aβ1-16 are increased and Aβ1-34 decreased following treatment with a γ-secretase inhibitor (GSI) (Portelius et al., 2010a, 2012), while treatment with GSM leads to an increase in Aβ1-37 together with decreases in Aβ1-39, Aβ1-40, and Aβ42 (Portelius et al., 2010b, 2014). Based on these previous studies and the immunoassay results of this study, the following peptides were preselected for quantification: Aβ1-14, Aβ1-15, Aβ1-16, Aβ1-34, Aβ1-37, Aβ1-38, Aβ1-39, and Aβ1-40. Aβ1-42 was deliberately excluded for analysis, as it was not reliably detected and quantifiable in all samples.

Treatment of TS21 neurons with selamectin (1.5 μM), the most potent of the avermectins, over 10 days resulted in an increase in Aβ1-37 and a decrease in Aβ1-40, with limited effects on Aβ1-14, Aβ1-15, and Aβ1-16 (Figures 4A–4C). An increase in Aβ1-37 and a decrease in Aβ1-40 is consistent with γ-secretase modulation (Portelius et al., 2010b), while a limited effect on Aβ1-14, Aβ1-15, and Aβ1-16 suggests minimal inhibition of γ-secretase function (Portelius et al., 2010a, 2012). It is noteworthy that the avermectin-induced increase in Aβ38 detected with the Aβ38/40/42 immunoassay was not detected with the IP-MALDI approach. This finding is not particularly unexpected, however; it has previously been shown that the GSM E2012 has no significant effect on Aβ38 by IP-MALDI (Portelius et al., 2010b), yet we detected a robust increase in Aβ38/Aβ42 with this compound using immunoassay detection (Figure 2B), largely driven by increases in Aβ38. While IP-MALDI is capable of detecting a wider range of Aβ peptides, it is not always as sensitive or quantitative as immunoassay approaches across all peptide species, particularly for low-abundance and relatively hydrophobic peptides.

The Effect of Avermectins on Aβ Production Is Independent of Their Known Pharmacology

The highest affinity targets for avermectins are invertebrate glutamate-gated chloride channels. Acting as irreversible agonists at these ion channels, avermectins stabilize the open conformation, admitting the flow of negatively charged chloride ions into cells, paralyzing nematodes and parasites (Wolstenholme and Rogers, 2005). Despite usual exclusion from the mammalian CNS, avermectins also have affinity for the mammalian ligand-gated chloride channels γ-aminobutyric $acid_A$ ($GABA_A$) and glycine, acting as positive allosteric modulators and direct partial agonists at nanomolar concentrations (Dawson et al., 2000; Shan et al., 2001). To determine whether the effects on Aβ production were due to activity at $GABA_A$ and/or glycine receptors, we assessed whether the effects of avermectins could be phenocopied or antagonized with specific agonists and antagonists of these receptors, respectively (Figure 5).

The effects of an ascending concentration of the $GABA_A$ receptor antagonist picrotoxin (0.3–100 μM) on the Aβ38/Aβ42 ratio were tested in the absence or presence of the two most potent avermectins identified in this system; ivermectin (1 μM) and selamectin (1 μM) (Figure 5A). Two-way

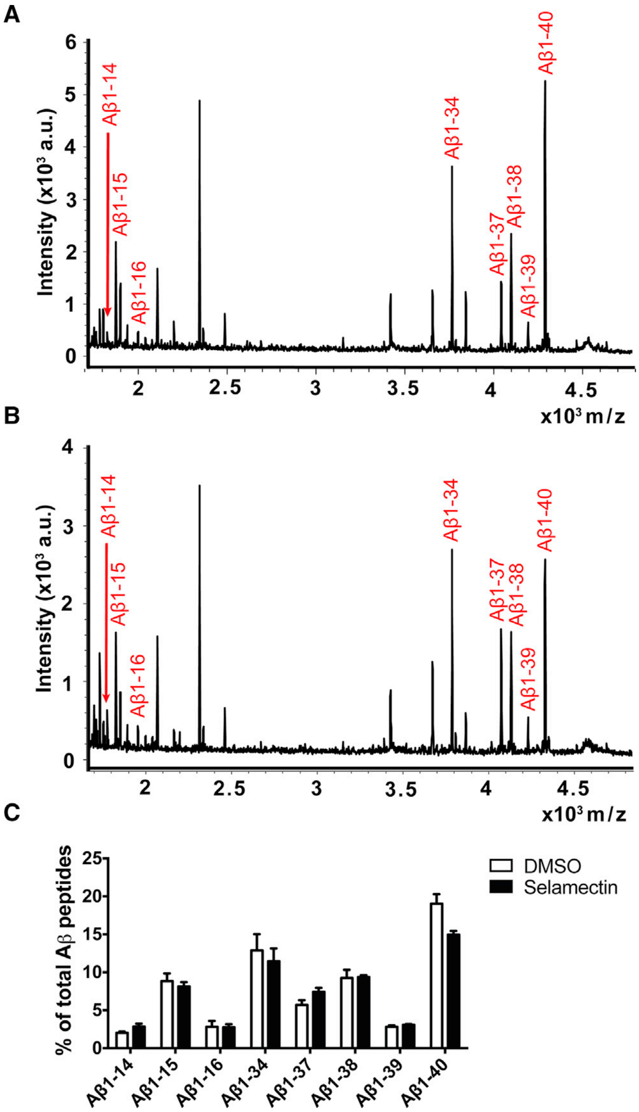

Figure 4. Selamectin Has Complex Effects on Aβ Peptide Production as Measured with IP-MALDI

(A and B) Representative Aβ IP-MALDI traces after treatment with DMSO vehicle (A) and selamectin (B). Peptides selected for quantitative analysis are highlighted in red.

(C) Analysis of peak areas of selected peptides as a percentage of total peak areas indicates an increase in Aβ1-37 and a decrease in Aβ1-40 following selamectin treatment.

n = 4 cultures per treatment. Error bars represent SD.

ANOVA revealed significant main effects of the avermectins ($F_{(2, 41)} = 176.8$, $p < 0.0001$) and picrotoxin ($F_{(5, 41)} = 9.996$, $p < 0.0001$). Crucially, however, there was no significant interaction between picrotoxin and the avermectins ($F_{(10, 41)} = 0.8645$, $p = 0.5724$). Additionally the potent $GABA_A$ receptor agonist muscimol (Figure 5B) had no effect on the Aβ38/Aβ42 ratio when tested up to 100 μM ($F_{(6, 17)} = 2.007$, $p = 0.1209$).

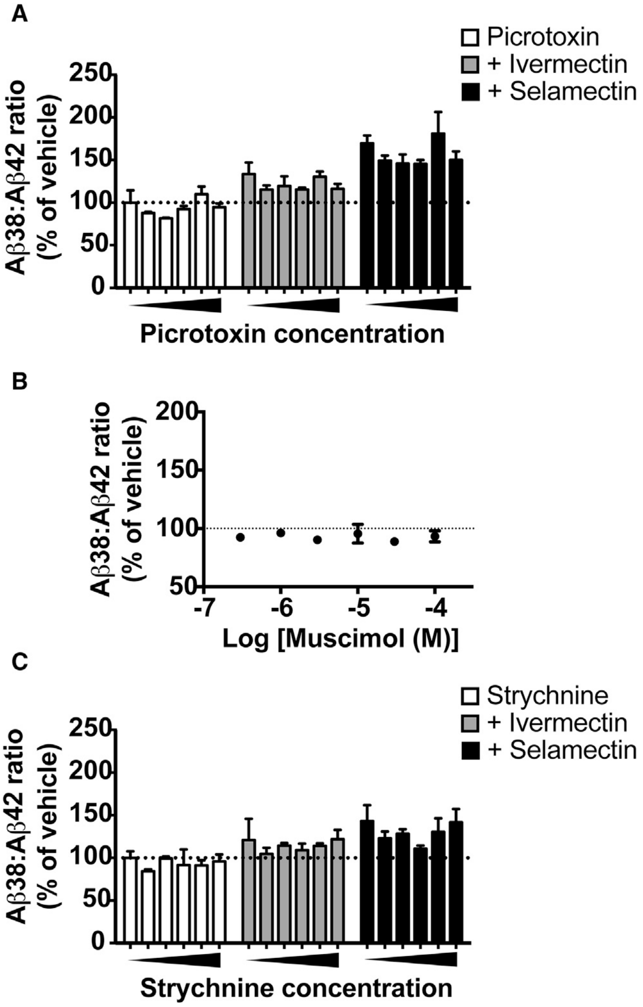

Figure 5. Avermectins Do Not Influence Aβ Processing via Their Known Pharmacological Targets

(A and B) The effects of ivermectin and selamectin are not blocked by the $GABA_A$ receptor antagonist picrotoxin (A) and are not phenocopied by the $GABA_A$ receptor agonist muscimol (B).

(C) Likewise, the glycine receptor antagonist strychnine exhibited no dose-dependent antagonism of avermectin effects of Aβ processing (see also Figure S1).

n = 3–6 cultures/treatment. Error bars represent SD.

Similarly, two-way ANOVA of ascending concentrations of the glycine receptor antagonist strychnine (0.3–30 μM) in the presence and absence of these avermectins (both tested at 1 μM) revealed a significant main effect of avermectins ($F_{(2, 41)} = 43.55$, $p < 0.0001$) and strychnine ($F_{(5, 41)} = 4.055$, $p = 0.0044$), but again no significant interaction between strychnine and the avermectins ($F_{(10, 41)} = 0.6173$, $p = 0.7901$). In addition, no inhibition of the concentration-dependent effect of ivermectin or selamectin on the Aβ38/Aβ42 ratio is observed when dose response is performed in the presence of strychnine (Figure S1).

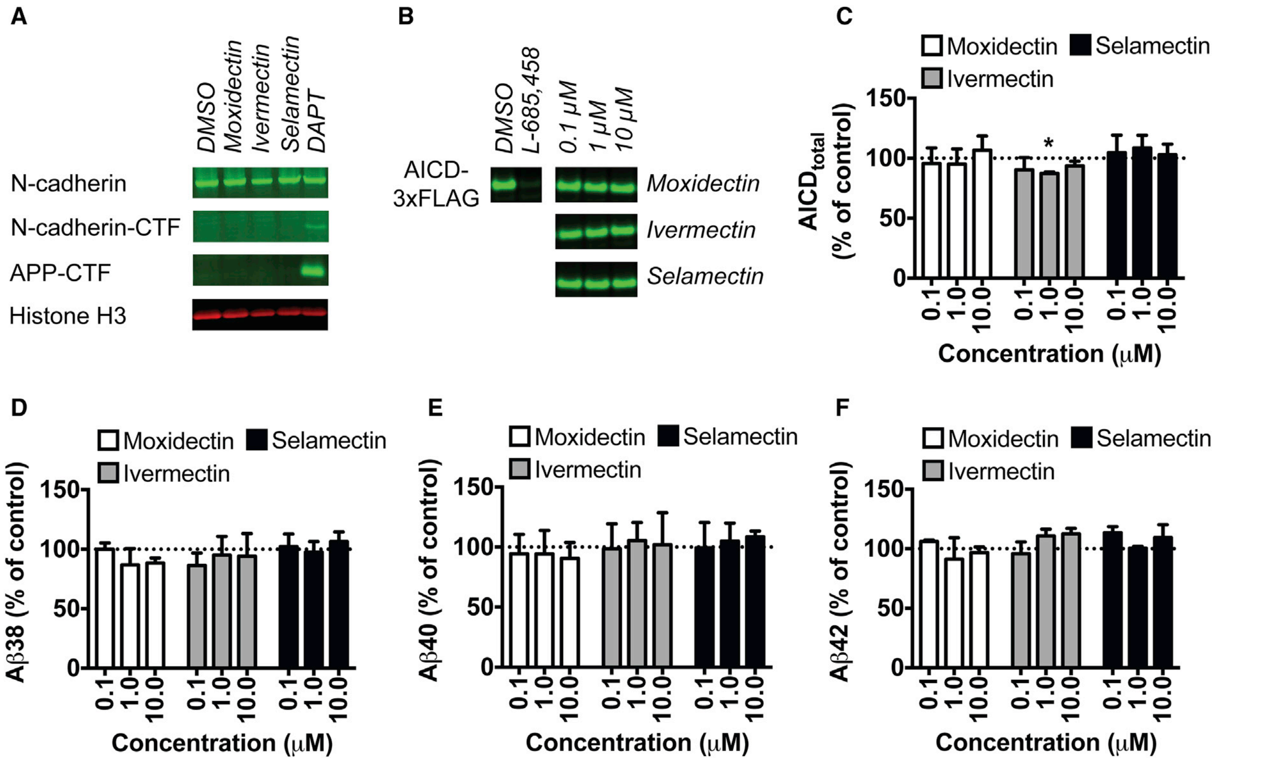

Figure 6. Avermectins Do Not Cause Accumulation of C-terminal Fragments of γ-Secretase Substrates, and Their Effects on Aβ Are Not Due to Direct Action on the Core γ-Secretase Complex

(A) In human cortical neurons, treatment with the conventional GSI DAPT causes significant accumulation of C-terminal fragments of the γ-secretase substrates APP and N-cadherin, whereas treatment with the milbemycin moxidectin or the avermectins ivermectin or selamectin does not (see also Figure S2).

(B) In a cell-free γ-secretase assay, the conventional GSI L-685,458 inhibits cleavage of APP C-terminal fragment by γ-secretase, and reduces the production of AICD.

(C) Moxidectin and the avermectins have no consistent effects, although 1 μM ivermectin causes a significant reduction of AICD production (C).

(D–F) In the same assay, milbemycins and avermectins induce no dose-dependent changes in the production of Aβ38 (D), Aβ40 (E), or Aβ42 (F) over DMSO control.

Western blots in (A) and (B) are representative of two to four cultures per treatment (A) and three independent experiments (B), respectively, and data shown in (C) to (F) are from three independent experiments. Error bars represent SD. *$p < 0.05$, Holm-Šídák adjusted one-sample t test versus 100% control value.

These results do not support the hypothesis that the avermectins are working via their known mammalian targets, the $GABA_A$ or glycine receptors, to affect Aβ processing.

Avermectins Do Not Cause Accumulation of γ-Secretase Substrates, and Do Not Directly Interact with the γ-Secretase Complex to Effect Changes in Aβ Production

One of the positive aspects of existing GSMs is that they do not cause the accumulation of the C-terminal fragments of APP or other γ-secretase substrates that is commonly observed following γ-secretase inhibition. Analysis of drug-treated TS21 cortical neurons revealed that, while the conventional GSI DAPT (1 μM) causes accumulation of APP and N-cadherin C-terminal fragments, neither moxidectin (10 μM) nor the avermectins ivermectin (1.5 μM) or selamectin (1.5 μM) had the same effect (Figure 6A), suggesting little inhibition of γ-secretase function. Chronic treatment over 30 days with the same compounds had similar effects, although ivermectin caused a mild accumulation of N-cadherin C-terminal fragment over this protracted treatment time (Figure S2).

A cell-free assay was performed to test whether avermectins act directly on the core γ-secretase complex to effect changes in Aβ processing (Figures 6B–6F). When C99-3×FLAG is introduced as a substrate in this system, efficiency of endopeptidase cleavage is assessed via the production of APP intracellular domain (AICD)-3×FLAG

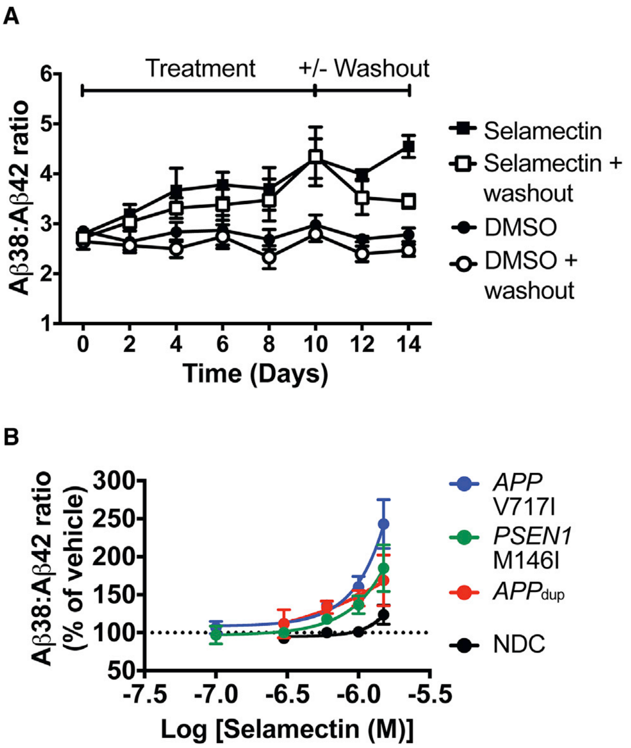

Figure 7. The Effect of Selamectin on Aβ Production in Cortical Neurons Is Reversible and Not Specific to the TS21 Genotype

(A) After 10 days of compound treatment, withdrawal of selamectin leads to a reduction in effect compared with continued treatment over 4 days (A).

(B) The effect of selamectin on Aβ processing is reproduced in neurons differentiated from patients carrying *APP* or *PSEN1* mutations, indicating that the effect is not specific to the TS21 genotype (B).

n = 3–6 cultures/treatment. Error bars represent SD.

measured by western blot, while the downstream production of Aβ species by sequential carboxypeptidase activity is measured by immunoassay (Chavez-Gutierrez et al., 2012; Szaruga et al., 2015). We utilized the prototypical γ-secretase inhibitor L-685,458 as a positive control for inhibited endopeptidase cleavage, which is reflected by a reduced production of AICD-3×FLAG (Figure 6B). The inactive milbemycin moxidectin and the two avermectins with the most potent effects on Aβ processing in a whole-cell system, selamectin and ivermectin, exhibited no significant dose-dependent effects on AICD-3×FLAG production (Figures 6B and 6C), although ivermectin exhibited a significant 10% reduction in AICD production at 1 μM ($t_{(2)} = 18.54$; $p = 0.0258$). Likewise, none of the macrocyclic lactone compounds had any appreciable effect on Aβ38 (Figure 6D), Aβ40 (Figure 6E), or Aβ42 (Figure 6F) production in this cell-free system (all one-sample t tests or Wilcoxon signed-rank tests $p > 0.05$ compared with 100% control values), despite strong effects in a whole-cell system at lower concentrations (see above). These findings indicate that the effects of avermectins on Aβ processing are not via direct action on the core γ-secretase complex.

Avermectin Effects on Aβ Production Are Reversible and Not Specific to TS21 Neurons

To determine whether the effects of selamectin on Aβ production were reversible, we performed a washout experiment whereby exposure to drug was discontinued after chronic treatment. After 10 days of continuous exposure, DMSO or 1.5 μM selamectin treatment was either continued or, in the washout condition, withdrawn and replaced with neural cell-culture medium alone (Figure 7A). Over the course of 10 days, selamectin increased the Aβ38/Aβ42 ratio compared with DMSO vehicle treatment. When treatment was withdrawn, however, the Aβ38/Aβ42 ratio in selamectin-treated neurons returned almost to vehicle-treated levels over the course of 4 days. These observations suggest that the effect of selamectin is at least partially reversible over this timescale.

The overexpression of genes on chromosome 21 other than *APP* is thought to contribute to the AD-like pathology observed in Down syndrome (Wiseman et al., 2015). To determine whether the effect of avermectins on Aβ production was specific to the TS21 genotype, or whether avermectins could alter APP processing in other genetic forms of AD, we tested selamectin in cortical neurons derived from iPSCs from a healthy control (non-diseased control [NDC]), and patients carrying an *APP* duplication (APP_{dup}), an *APP* missense mutation (*APP* V717I), or a Presenilin-1 mutation (*PSEN1* M146I) (Figure 7B).

Selamectin increased the ratio of Aβ38 to Aβ42 in a dose-dependent manner in neurons from patients carrying APP_{dup} ($F_{(4,\ 12)} = 8.741$, $p = 0.0015$), *APP* V717I ($F_{(5,\ 12)} = 38.09$, $p < 0.0001$), and *PSEN1* M146I ($F_{(5,\ 15)} = 16.8$, $p < 0.0001$) mutations, but not in neurons from a non-diseased background ($H_{(4)} = 9.208$, $p = 0.0561$). This difference is likely due to the fact that data from the non-diseased control line were not normally distributed and were analyzed non-parametrically, as using the more powerful parametric analysis does reveal an effect in this genetic background ($F_{(4,\ 12)} = 5.532$, $p = 0.0092$). These findings together suggest that the effect of the avermectins is generalizable, and not limited to the TS21 genotype or reliant upon increase in *APP* copy numbers and protein levels.

DISCUSSION

We report here the identification, by phenotypic screening in a human stem cell model of AD, of γ-secretase-independent modulators of APP processing. A class of anthelmintic

macrocyclic lactones, the avermectins, increases the relative production of short forms of Aβ and reduces the relative production of longer Aβ fragments in human cortical neurons. This effect is independent of the known molecular targets of avermectins in the mammalian nervous system and, moreover, despite an effect phenotypically similar to that of existing GSMs, is not due to a direct interaction with the core γ-secretase complex. The avermectins phenocopy γ-secretase modulation in healthy control neurons and multiple different models of genetic forms of AD, including TS21, *APP* duplication, and *PSEN1* and *APP* mutations. In addition to the discovery of avermectins as modifiers of Aβ peptide production, this study demonstrates the utility of human stem cell models for small-molecule phenotypic screens of disease-relevant biology in neurodegeneration. Future investigation will focus on elucidating the mechanisms and molecular targets behind this phenomenon.

The effect of the avermectins on Aβ production mimics that of GSMs, a diverse class of compounds that, either through action on γ-secretase or APP, alters APP cleavage in favor of shorter Aβ peptides. With a phenotypic effect opposite to that of the *PSEN1* mutations leading to early-onset fAD, which drives an accumulation of longer Aβ peptides due to impaired carboxypeptidase cleavage efficiency of γ-secretase (Chavez-Gutierrez et al., 2012), the promise of GSMs lies in their ability to reduce the accumulation of toxic longer forms of Aβ while preserving γ-secretase cleavage of its other substrates. Of note, the very first GSMs were NSAIDs, whose effect on increasing Aβ38 while reducing Aβ42 production was first discovered thorough a phenotypic screen in transfected cells and ascribed to a mechanism independent of their previously known molecular targets (Weggen et al., 2001). Target identification of the early GSMs has led to more potent and selective derivatives, a number of which are currently in human trials for AD (Hall and Patel, 2014). We have demonstrated that the avermectins elicit changes in Aβ production that are comparable with those in existing GSMs, and similarly avoid accumulation of γ-secretase substrates, but act through a γ-secretase-independent pathway. It is hoped that target identification in future studies may lead to derivatives with higher potency, in addition to more favorable physicochemical and pharmacokinetic properties than the existing avermectins, which are highly lipophilic (Prichard et al., 2012) and poorly CNS penetrant (Schinkel et al., 1994).

While the results of the membrane-based γ-secretase assays are not consistent with a direct effect on the core γ-secretase complex, there are indirect ways by which APP proteolysis may be altered that could go undetected in a cell-free assay. The specific APP processing paths leading to the generation of different Aβ species are determined by the dynamic shuttling between intracellular compartments of the membrane-bound substrates and the secretases, which must co-localize for proteolysis to occur (see review by Small and Gandy, 2006). These shuttling processes are amenable to pharmacological intervention, and indeed it was recently shown that small-molecule stabilization of the neuronal retromer complex, which traffics APP from endosomes to the Golgi, limits co-localization of APP and β-secretase in the endosomal membrane, thus causing a reduction in total Aβ peptide production (Mecozzi et al., 2014).

More broadly, this study confirms the feasibility of unbiased phenotypic drug screening for modifiers of neurological disease in patient-derived neurons; an approach that has been a core aim of this technology since its inception (Khurana et al., 2015). In the small number of phenotypic drug screens in human stem cell models of neurodegeneration reported thus far, small molecules have been identified which rescued downregulated proteins in familial dysautonomia (Lee et al., 2012) and inhibited aberrant protein aggregation in amyotrophic lateral sclerosis (Burkhardt et al., 2013). Cortical neurons derived from patients carrying fAD mutations or more complex forms of AD, including TS21, faithfully reproduce pathological changes in disease-relevant proteins reported in vivo, without the need for artificial overexpression or exogenous toxic insult (Israel et al., 2012; Moore et al., 2015; Shi et al., 2012b; Yagi et al., 2011). Not only has this system previously provided mechanistic insight into AD initiation and progression (Moore et al., 2015), but we have demonstrated here that the aberrant changes in Aβ production in AD human neurons provide a sensitized and relevant background for unbiased phenotypic screening, in a model system that is reproducible, scalable, and responsive to existing modulators of Aβ production. While cell-based phenotypic screens have traditionally utilized immortalized cell lines or overexpression systems, the ability to derive disease-relevant cell types from reprogrammed human cells is likely to be of additional benefit for phenotypic assays in neurodegenerative diseases, due to the high degree of cell-type- and cell-subtype-specific pathology (Mattson and Magnus, 2006).

The two main avenues for the discovery and development of new medicines are target-based screening and cell-based phenotypic screening. In the first, a molecular target of interest in the disease is defined and validated prior to the screen, whereas in the second the phenotype of interest is screened for modifiers of which their underlying molecular mechanism of action is not yet known. While early drug discovery was almost entirely based on screening against known phenotypes, more recent advances in genomics and improved understanding of the molecular mechanisms underlying disease have shifted the focus toward more target-based approaches. However, the complex and multifactorial nature of human disorders, particularly those of the CNS, has hampered identification of relevant and specific singular drug targets, and perhaps

explains why phenotypic screening still contributes to a greater proportion of first-in-class drugs (Swinney and Anthony, 2011). The lack of assumptions with a phenotypic approach also means there is the potential to uncover novel disease pathways. It has been argued that an initial approach using empirical phenotypic assays followed by hypothesis-driven target identification might provide an optimum combination of techniques for the identification and development of new treatments for complex human disorders (Swinney, 2013). Combined with the ability to derive disease-specific cell types from reprogrammed patient cells, phenotypic screening is once again in the spotlight as a powerful tool in the search for disease-modifying treatments.

Using cortical neurons derived from an individual with TS21, a common complex genetic form of AD, we demonstrated the feasibility of phenotypic, small-molecule screens in human stem cell models of AD. We identified the anthelminthic avermectins as modulators of Aβ production, which act independently of γ–secretase to alter APP processing in manner similar to γ–secretase modulation. The recent emergence and continued development of human cell models of disease in combination with traditional phenotypic screening approaches promises to allow the identification of potential drug candidates in addition to uncovering new pathways underlying disease pathology.

EXPERIMENTAL PROCEDURES

Cell Culture

Primary screening and mechanistic studies were performed on neurons differentiated from iPSCs generated from an individual with TS21 (Park et al., 2008). Additional genotypes were non-diseased controls (NDCs; Israel et al., 2012), *APP* duplication (*APP*$_{dup}$) (Israel et al., 2012), *APP* V717I (Moore et al., 2015), and *PSEN1* M146I (Moore et al., 2015). iPSCs were cultured and maintained feeder-free in Essential-8 (Life Technologies), without antibiotics. Directed differentiation of iPSCs to cortical neurons was performed as previously described (Shi et al., 2012a, 2012c). Neural stem/progenitor cells were produced by pooling neural inductions from each iPSC line. Pooling at this stage was carried out to minimize experimental variability among subsequent neuronal differentiations. Neural stem/progenitor cells were subsequently used for independent neural differentiations over 15–30 days in parallel to generate postmitotic cortical neurons and astrocytes for each experiment and/or drug treatment. Each separate neuronal differentiation was considered a biological replicate. Drug screening was performed on neurons in 96-well plates (Greiner and Ibidi), with some additional experiments performed in 12-well plates (Corning).

Immunocytochemistry

Cell cultures were fixed in 4% (w/v) paraformaldehyde in PBS and blocked with 5% normal donkey serum in 0.3% (v/v) Tween 20 in Tris-buffered saline before immunofluorescent staining. Primary antibodies used were anti-MAP2 (ab5392, Abcam), anti-CTIP2 (ab18465, Abcam), and anti-TBR1 (ab31940, Abcam), and secondary antibodies were Alexa Fluor conjugated. Stained cells were imaged on an Olympus FV1000 inverted confocal microscope and data imported into PerkinElmer Volocity for visualization.

Drugs

The Prestwick Chemical library of 1,200 Food and Drug Administration-approved compounds was supplied at a concentration of 10 mM in DMSO in a 96-well format (Prestwick Chemical). Additional compounds used included (R)-flurbiprofen (Cayman), the γ-secretase modulator E2012 (ChemExpress), abamectin (Santa Cruz Biotechnology), ivermectin (Tocris Biosciences), emamectin benzoate (Abcam), selamectin (MicroSource Discovery Systems), moxidectin (Santa Cruz), strychnine HCl (Abcam), picrotoxin (Tocris), muscimol (Tocris), and the γ-secretase inhibitors DAPT (Sigma) and L-685,458 (Merck-Millipore).

Drug Treatment

For the primary screen, drugs were applied at a final concentration of 1 μM in 0.1% DMSO in neural cell culture medium. In additional experiments, drugs were dissolved in DMSO or H_2O, and applied to cells such that final concentrations of DMSO did not exceed 0.4%. All drug treatments began 50–65 days after the initiation of neural induction, with media collected and refreshed at 48-hr intervals. Compound effects were normalized to appropriate vehicle controls within each plate.

Biochemical Assays

Conditioned medium for biochemical analysis was spun at 800 × *g* to remove cellular debris, and supernatant stored at −20°C until use. Measurement of Aβ38, Aβ40, and Aβ42 was performed by multiplexed immunoassay (Meso Scale Diagnostics), and measurement of LDH activity was performed using a Cytotoxicity Detection kit (Roche).

Immunoprecipitation and Matrix-Assisted Laser Desorption/Ionization

Conditioned medium for IP-MALDI analysis was centrifuged at 1,200 × *g*, and supernatant stored at −80°C in protein LoBind microcentrifuge tubes (Eppendorf) until use. The Aβ peptide profile was determined by immunoprecipitation, using Aβ-specific antibodies coupled to magnetic beads, in combination with a MALDI-TOF/TOF instrument (UltraFleXtreme, Bruker Daltonics) as described previously (Portelius et al., 2007). Samples were prepared as described previously (Portelius et al., 2007). Of the 25 Aβ species identified, it was determined that Aβ1-19 was highly variable between samples within the same treatment groups. As it has not been identified as a species of interest in previous studies investigating pharmacological modulation of secretase complexes, Aβ1-19 was excluded from further analysis and its contribution to total Aβ removed.

Immunoblotting

After collection in ice-cold PBS, cells were lysed in RIPA buffer containing 1 mM DTT, protease and phosphatase inhibitors (Pierce),

and 25 U/mL DNAse. The soluble fraction of cell lysates was subjected to SDS-PAGE and immunoblotting. Primary antibodies used were N-cadherin (610921, BD Transduction Laboratories), APP (SIG-39152, Covance), and histone H3 (ab1791, Abcam). Immunoblot detection was performed using the Odyssey Infrared Imaging System.

Cell-Free γ-Secretase Assays

The γ-secretase in vitro activity assay was performed as described previously (Szaruga et al., 2015), with minor modifications. See Supplemental Experimental Procedures for additional details.

Statistical Analysis

Statistical analysis was performed using IBM SPSS and GraphPad Prism. Alpha was set to 0.05 for all significance testing. n refers to the number of independent cultures of iPSC-derived neurons, except in the case of cell-free experiments where n represents independent experiments. See Supplemental Experimental Procedures for detailed statistical procedures.

SUPPLEMENTAL INFORMATION

Supplemental Information includes Supplemental Experimental Procedures and two figures and can be found with this article online at http://dx.doi.org/10.1016/j.stemcr.2017.02.006.

AUTHOR CONTRIBUTIONS

P.W.B., F.J.L., and J.S. conceptualized the study and key experiments. P.W.B. and J.S. collected and analyzed most of the experimental data. E.P., H.K., K.B., and H.Z. performed IP-MALDI experiments. L.S. and B.d.S. performed cell-free experiments. P.W.B., F.J.L., and J.S. wrote the manuscript. All authors edited and approved the final manuscript.

ACKNOWLEDGMENTS

P.W.B. received funding through the Alborada Trust's support of the Alzheimer's Research UK Stem Cell Research Centre. J.S. was supported by the Innovative Medicines Initiative Consortium, StemBANCC (grant no, 115439). H.Z. was supported by the Swedish Research Council (grant no: 2013-2546) and the European Research Council (grant no: 681712). F.J.L. is a Wellcome Trust Senior Investigator (grant no. 101052/2/13/2) and gratefully acknowledges the support of the Alborada Trust and Alzheimer's Research UK (grant no. ARUK-SCRC 2014-1). Research in the Gurdon Institute benefits from core support from the Wellcome Trust and Cancer Research UK. The authors thank Dr. Steven Moore for provision of technical support and advice.

Received: September 22, 2016
Revised: February 7, 2017
Accepted: February 8, 2017
Published: March 9, 2017

REFERENCES

Boutros, M., Bras, L.P., and Huber, W. (2006). Analysis of cell-based RNAi screens. Genome Biol. *7*, R66.

Brideau, C., Gunter, B., Pikounis, B., and Liaw, A. (2003). Improved statistical methods for hit selection in high-throughput screening. J. Biomol. Screen. *8*, 634–647.

Burkhardt, M.F., Martinez, F.J., Wright, S., Ramos, C., Volfson, D., Mason, M., Garnes, J., Dang, V., Lievers, J., Shoukat-Mumtaz, U., et al. (2013). A cellular model for sporadic ALS using patient-derived induced pluripotent stem cells. Mol. Cell Neurosci. *56*, 355–364.

Chavez-Gutierrez, L., Bammens, L., Benilova, I., Vandersteen, A., Benurwar, M., Borgers, M., Lismont, S., Zhou, L., Van Cleynenbreugel, S., Esselmann, H., et al. (2012). The mechanism of gamma-Secretase dysfunction in familial Alzheimer disease. EMBO J. *31*, 2261–2274.

Dawson, G.R., Wafford, K.A., Smith, A., Marshall, G.R., Bayley, P.J., Schaeffer, J.M., Meinke, P.T., and McKernan, R.M. (2000). Anticonvulsant and adverse effects of avermectin analogs in mice are mediated through the gamma-aminobutyric acid(A) receptor. J. Pharmacol. Exp. Ther. *295*, 1051–1060.

Ebert, A.D., Yu, J., Rose, F.F., Jr., Mattis, V.B., Lorson, C.L., Thomson, J.A., and Svendsen, C.N. (2009). Induced pluripotent stem cells from a spinal muscular atrophy patient. Nature *457*, 277–280.

Ertekin-Taner, N. (2007). Genetics of Alzheimer's disease: a centennial review. Neurol. Clin. *25*, 611–667, v.

Findeis, M.A. (2007). The role of amyloid beta peptide 42 in Alzheimer's disease. Pharmacol. Ther. *116*, 266–286.

Fisher, M.H., and Mrozik, H. (1992). The chemistry and pharmacology of avermectins. Annu. Rev. Pharmacol. Toxicol. *32*, 537–553.

Hall, A., and Patel, T.R. (2014). γ-Secretase modulators: current status and future directions. Prog. Med. Chem. *53*, 101–145.

Hardy, J., and Allsop, D. (1991). Amyloid deposition as the central event in the aetiology of Alzheimer's disease. Trends Pharmacol. Sci. *12*, 383–388.

Hardy, J.A., and Higgins, G.A. (1992). Alzheimer's disease: the amyloid cascade hypothesis. Science *256*, 184–185.

Inglese, J., Johnson, R.L., Simeonov, A., Xia, M., Zheng, W., Austin, C.P., and Auld, D.S. (2007). High-throughput screening assays for the identification of chemical probes. Nat. Chem. Biol. *3*, 466–479.

Israel, M.A., Yuan, S.H., Bardy, C., Reyna, S.M., Mu, Y., Herrera, C., Hefferan, M.P., Van Gorp, S., Nazor, K.L., Boscolo, F.S., et al. (2012). Probing sporadic and familial Alzheimer's disease using induced pluripotent stem cells. Nature *482*, 216–220.

Khurana, V., Tardiff, D.F., Chung, C.Y., and Lindquist, S. (2015). Toward stem cell-based phenotypic screens for neurodegenerative diseases. Nat. Rev. Neurol. *11*, 339–350.

Kuperstein, I., Broersen, K., Benilova, I., Rozenski, J., Jonckheere, W., Debulpaep, M., Vandersteen, A., Segers-Nolten, I., Van Der Werf, K., Subramaniam, V., et al. (2010). Neurotoxicity of Alzheimer's disease Abeta peptides is induced by small changes in the Abeta42 to Abeta40 ratio. EMBO J. *29*, 3408–3420.

Lee, G., Ramirez, C.N., Kim, H., Zeltner, N., Liu, B., Radu, C., Bhinder, B., Kim, Y.J., Choi, I.Y., Mukherjee-Clavin, B., et al. (2012). Large-scale screening using familial dysautonomia induced

pluripotent stem cells identifies compounds that rescue IKBKAP expression. Nat. Biotechnol. *30*, 1244–1248.

Mattson, M.P., and Magnus, T. (2006). Ageing and neuronal vulnerability. Nat. Rev. Neurosci. *7*, 278–294.

Mecozzi, V.J., Berman, D.E., Simoes, S., Vetanovetz, C., Awal, M.R., Patel, V.M., Schneider, R.T., Petsko, G.A., Ringe, D., and Small, S.A. (2014). Pharmacological chaperones stabilize retromer to limit APP processing. Nat. Chem. Biol. *10*, 443–449.

Moore, S., Evans, L.D., Andersson, T., Portelius, E., Smith, J., Dias, T.B., Saurat, N., McGlade, A., Kirwan, P., Blennow, K., et al. (2015). APP metabolism regulates tau proteostasis in human cerebral cortex neurons. Cell Rep. *11*, 689–696.

Park, I.H., Arora, N., Huo, H., Maherali, N., Ahfeldt, T., Shimamura, A., Lensch, M.W., Cowan, C., Hochedlinger, K., and Daley, G.Q. (2008). Disease-specific induced pluripotent stem cells. Cell *134*, 877–886.

Portelius, E., Tran, A.J., Andreasson, U., Persson, R., Brinkmalm, G., Zetterberg, H., Blennow, K., and Westman-Brinkmalm, A. (2007). Characterization of amyloid beta peptides in cerebrospinal fluid by an automated immunoprecipitation procedure followed by mass spectrometry. J. Proteome Res. *6*, 4433–4439.

Portelius, E., Dean, R.A., Gustavsson, M.K., Andreasson, U., Zetterberg, H., Siemers, E., and Blennow, K. (2010a). A novel Abeta isoform pattern in CSF reflects gamma-secretase inhibition in Alzheimer disease. Alzheimers Res. Ther. *2*, 7.

Portelius, E., Van Broeck, B., Andreasson, U., Gustavsson, M.K., Mercken, M., Zetterberg, H., Borghys, H., and Blennow, K. (2010b). Acute effect on the Abeta isoform pattern in CSF in response to gamma-secretase modulator and inhibitor treatment in dogs. J. Alzheimers Dis. *21*, 1005–1012.

Portelius, E., Zetterberg, H., Dean, R.A., Marcil, A., Bourgeois, P., Nutu, M., Andreasson, U., Siemers, E., Mawuenyega, K.G., Sigurdson, W.C., et al. (2012). Amyloid-beta(1-15/16) as a marker for gamma-secretase inhibition in Alzheimer's disease. J. Alzheimers Dis. *31*, 335–341.

Portelius, E., Appelkvist, P., Stromberg, K., and Hoglund, K. (2014). Characterization of the effect of a novel gamma-secretase modulator on Abeta: a clinically translatable model. Curr. Pharm. Des. *20*, 2484–2490.

Prichard, R., Menez, C., and Lespine, A. (2012). Moxidectin and the avermectins: consanguinity but not identity. Int. J. Parasitol. Drugs Drug Resist. *2*, 134–153.

Rovelet-Lecrux, A., Hannequin, D., Raux, G., Le Meur, N., Laquerriere, A., Vital, A., Dumanchin, C., Feuillette, S., Brice, A., Vercelletto, M., et al. (2006). APP locus duplication causes autosomal dominant early-onset Alzheimer disease with cerebral amyloid angiopathy. Nat. Genet. *38*, 24–26.

Rumble, B., Retallack, R., Hilbich, C., Simms, G., Multhaup, G., Martins, R., Hockey, A., Montgomery, P., Beyreuther, K., and Masters, C.L. (1989). Amyloid A4 protein and its precursor in Down's syndrome and Alzheimer's disease. N. Engl. J. Med. *320*, 1446–1452.

Sanchez-Danes, A., Richaud-Patin, Y., Carballo-Carbajal, I., Jimenez-Delgado, S., Caig, C., Mora, S., Di Guglielmo, C., Ezquerra, M., Patel, B., Giralt, A., et al. (2012). Disease-specific phenotypes in dopamine neurons from human iPS-based models of genetic and sporadic Parkinson's disease. EMBO Mol. Med. *4*, 380–395.

Schinkel, A.H., Smit, J.J., van Tellingen, O., Beijnen, J.H., Wagenaar, E., van Deemter, L., Mol, C.A., van der Valk, M.A., Robanus-Maandag, E.C., te Riele, H.P., et al. (1994). Disruption of the mouse mdr1a P-glycoprotein gene leads to a deficiency in the blood-brain barrier and to increased sensitivity to drugs. Cell *77*, 491–502.

Shan, Q., Haddrill, J.L., and Lynch, J.W. (2001). Ivermectin, an unconventional agonist of the glycine receptor chloride channel. J. Biol. Chem. *276*, 12556–12564.

Shi, Y., Kirwan, P., and Livesey, F.J. (2012a). Directed differentiation of human pluripotent stem cells to cerebral cortex neurons and neural networks. Nat. Protoc. *7*, 1836–1846.

Shi, Y., Kirwan, P., Smith, J., MacLean, G., Orkin, S.H., and Livesey, F.J. (2012b). A human stem cell model of early Alzheimer's disease pathology in Down syndrome. Sci. Transl. Med. *4*, 124ra129.

Shi, Y., Kirwan, P., Smith, J., Robinson, H.P., and Livesey, F.J. (2012c). Human cerebral cortex development from pluripotent stem cells to functional excitatory synapses. Nat. Neurosci. *15*, 477–486, S471.

Sleegers, K., Brouwers, N., Gijselinck, I., Theuns, J., Goossens, D., Wauters, J., Del-Favero, J., Cruts, M., van Duijn, C.M., and Van Broeckhoven, C. (2006). APP duplication is sufficient to cause early onset Alzheimer's dementia with cerebral amyloid angiopathy. Brain *129*, 2977–2983.

Small, S.A., and Gandy, S. (2006). Sorting through the cell biology of Alzheimer's disease: intracellular pathways to pathogenesis. Neuron *52*, 15–31.

Soares, H.D., Gasior, M., Toyn, J.H., Wang, J.S., Hong, Q., Berisha, F., Furlong, M.T., Raybon, J., Lentz, K.A., Sweeney, F., et al. (2016). The gamma secretase modulator, BMS-932481, modulates Abeta peptides in the plasma and CSF of healthy volunteers. J. Pharmacol. Exp. Ther. *358*, 138–150.

Swinney, D.C. (2013). Phenotypic vs. target-based drug discovery for first-in-class medicines. Clin. Pharmacol. Ther. *93*, 299–301.

Swinney, D.C., and Anthony, J. (2011). How were new medicines discovered? Nat. Rev. Drug Discov. *10*, 507–519.

Szaruga, M., Veugelen, S., Benurwar, M., Lismont, S., Sepulveda-Falla, D., Lleo, A., Ryan, N.S., Lashley, T., Fox, N.C., Murayama, S., et al. (2015). Qualitative changes in human gamma-secretase underlie familial Alzheimer's disease. J. Exp. Med. *212*, 2003–2013.

Takahashi, K., and Yamanaka, S. (2006). Induction of pluripotent stem cells from mouse embryonic and adult fibroblast cultures by defined factors. Cell *126*, 663–676.

Takahashi, K., Tanabe, K., Ohnuki, M., Narita, M., Ichisaka, T., Tomoda, K., and Yamanaka, S. (2007). Induction of pluripotent stem cells from adult human fibroblasts by defined factors. Cell *131*, 861–872.

Takami, M., Nagashima, Y., Sano, Y., Ishihara, S., Morishima-Kawashima, M., Funamoto, S., and Ihara, Y. (2009). gamma-Secretase: successive tripeptide and tetrapeptide release from the transmembrane domain of beta-carboxyl terminal fragment. J. Neurosci. *29*, 13042–13052.

Toyn, J.H., Boy, K.M., Raybon, J., Meredith, J.E., Roberston, A.S., Guss, V., Hoque, N., Sweeney, F., Zhuo, X., Clarke, W., et al. (2016). Robust translation of GSM pharmacology across preclinical species and human subjects. J. Pharmacol. Exp. Ther. *358*, 125–137.

Weggen, S., Eriksen, J.L., Das, P., Sagi, S.A., Wang, R., Pietrzik, C.U., Findlay, K.A., Smith, T.E., Murphy, M.P., Bulter, T., et al. (2001). A subset of NSAIDs lower amyloidogenic Abeta42 independently of cyclooxygenase activity. Nature *414*, 212–216.

Wiseman, F.K., Al-Janabi, T., Hardy, J., Karmiloff-Smith, A., Nizetic, D., Tybulewicz, V.L., Fisher, E.M., and Strydom, A. (2015). A genetic cause of Alzheimer disease: mechanistic insights from Down syndrome. Nat. Rev. Neurosci. *16*, 564–574.

Wolstenholme, A.J., and Rogers, A.T. (2005). Glutamate-gated chloride channels and the mode of action of the avermectin/milbemycin anthelmintics. Parasitology *131*(Suppl), S85–S95.

Yagi, T., Ito, D., Okada, Y., Akamatsu, W., Nihei, Y., Yoshizaki, T., Yamanaka, S., Okano, H., and Suzuki, N. (2011). Modeling familial Alzheimer's disease with induced pluripotent stem cells. Hum. Mol. Genet. *20*, 4530–4539.

Yu, Y., Logovinsky, V., Schuck, E., Kaplow, J., Chang, M.K., Miyagawa, T., Wong, N., and Ferry, J. (2014). Safety, tolerability, pharmacokinetics, and pharmacodynamics of the novel gamma-secretase modulator, E2212, in healthy human subjects. J. Clin. Pharmacol. *54*, 528–536.

Stem Cell Reports, Volume *8*

Supplemental Information

Phenotypic Screening Identifies Modulators of Amyloid Precursor Protein Processing in Human Stem Cell Models of Alzheimer's Disease

Philip W. Brownjohn, James Smith, Erik Portelius, Lutgarde Serneels, Hlin Kvartsberg, Bart De Strooper, Kaj Blennow, Henrik Zetterberg, and Frederick J. Livesey

Supplemental Information

Supplemental Data

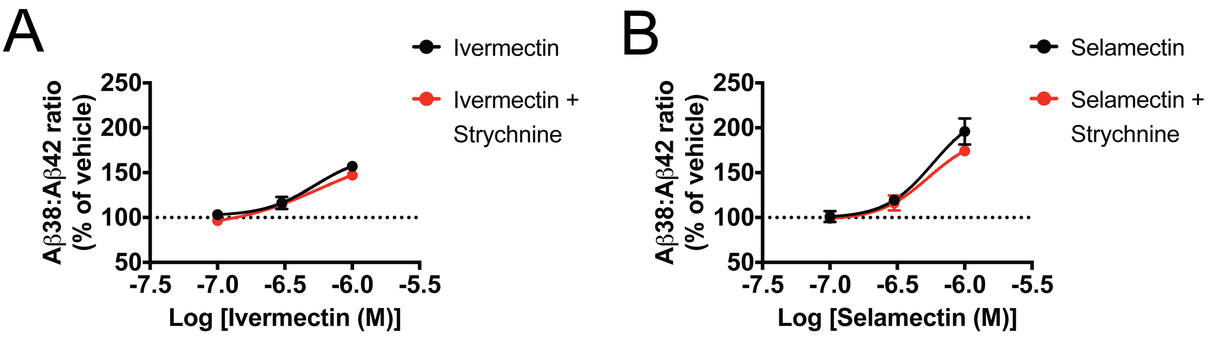

Figure S1. Avermectins do not alter Aβ production in human neurons via activation of glycine receptors. Ivermectin (A) and selamectin (B) dose dependently increase the ratio of Aβ38 to Aβ42 in the presence and absence of the glycine receptor antagonist strychnine (30 µM). n = 3 cultures/treatment. Error bars represent standard deviations.

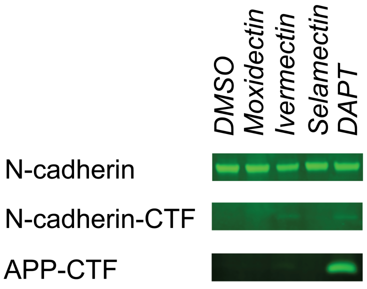

Figure S2. Chronic treatment with avermectins does not cause significant accumulation of γ-secretase substrate CTFs in human neurons. Treatment of human neurons for 30 days with DAPT results in mild accumulation of N-cadherin-CTF, and significant accumulation of APP-CTF. In contrast, 30 days of treatment with moxidectin or the avermectins ivermectin and selamectin causes no significant accumulation of N-cadherin-CTF or APP-CTF, while ivermectin results in mild accumulation of N-cadherin-CTF. Western blots are representative of 2 – 3 cultures/treatment.

Supplemental Experimental Procedures

Cell-free γ-secretase assays

In vitro reactions with 150 ng/µl DRM γ-secretase from Sf9 cells expressing human PSEN1-APH1A-NCT-PEN2 γ-secretase and 1.2 µM APP-C99–3×FLAG were performed in 0.3% CHAPSO and 2.5% DMSO or compound at the indicated concentration for 1 h at 37 °C. To detect AICD the substrate was extracted by adding 1 volume chloroform/methanol (2:1, v/v). Then, the aqueous fraction (AICD Products) was taken and subjected to SDS-PAGE and quantitative western immunoblot. AICD-3×FLAG was determined with the anti-FLAG M2 (Sigma) and goat anti-mouse IR800 (Pierce) antibodies. Infrared signals were detected and quantified using the Odyssey Infrared Imaging System. To determine de novo production of Aβ peptides, Aβ38, Aβ40, and Aβ42 levels in reactions were quantified on Multi-Spot 96-well plates precoated with anti-Aβ38, -Aβ40, and -Aβ42 antibodies and detected with SULFO-TAG JRF/AβN/25 using multiplex MSD technology (Meso Scale Discovery). The anti-Aβ antibodies were generously provided by Mark Mercken from Janssen Pharmaceutica.

Statistical analysis

Normality was probed with the Shapiro-Wilk normality test and by visualization of Q-Q plots. Dose-response data were analyzed with parametric one-way ANOVA when normally distributed, or Kruskall-Wallis H test when not. For antagonism studies, data were analyzed by two-way ANOVA. While small deviations from

normality could not be rectified with transformation in this case, parametric analysis was still performed in order to obtain the most statistical power, with the caveat that the type-I error rate was likely to be inflated. Cell-free assays were analyzed by corrected one-sample t-tests or Wilcoxin signed rank tests. Multiple testing was corrected using the Holm-Šídák method where appropriate.
